# The Development and Validation of a Measure of Mental Health, Help-Seeking Beliefs in Arabic-Speaking Refugees

**DOI:** 10.1177/10731911231220482

**Published:** 2023-12-30

**Authors:** Natalie Mastrogiovanni, Yulisha Byrow, Angela Nickerson

**Affiliations:** 1 University of New South Wales, Sydney, Australia

**Keywords:** refugees, posttraumatic stress disorder (PTSD), help-seeking, beliefs, stigma

## Abstract

Despite reporting elevated rates of posttraumatic stress disorder (PTSD), refugees are less likely than other groups to seek psychological treatment. Relatively little attention has been paid to the role of negative help-seeking beliefs in influencing treatment uptake. The current study sought to develop and psychometrically validate a novel measure indexing negative help-seeking beliefs for refugees (Help-Seeking Beliefs Scale [HSBS]). In this study, 262 Arabic-speaking refugee participants completed an online survey consisting of the HSBS along with measures indexing similar constructs (self-stigma of PTSD and help-seeking, perceived stigma, negative help-seeking attitudes, and help-seeking intentions). Factor analysis revealed a three-factor structure aligning with key themes identified in the literature: (a) Fear of Negative Consequences, (b) Inappropriateness, and (c) Perceived Necessity. The scale demonstrated excellent internal consistency, convergent validity, and predicted reduced help-seeking intentions. Results support the utility of a novel measure capturing a prominent help-seeking barrier in a population with high psychopathology and low treatment uptake.

Refugees have often been exposed to a wide variety of traumatic events in their country of origin, including torture, violence, and imprisonment (Schlaudt et al., 2020). Accordingly, rates of posttraumatic stress disorder (PTSD) are elevated among refugees compared with the general population even after long-term resettlement (Blackmore et al., 2020; Bogic et al., 2015). Despite the high prevalence of psychopathology among people from refugee backgrounds (referred to as refugees for parsimony), studies consistently report that only a small percentage of refugees seek mental health treatment compared with the general population (Fuhr et al., 2019; Satinsky et al., 2019; Slewa-Younan et al., 2015). This discrepancy between needs and service utilization creates a treatment gap, presenting a critical challenge to supporting refugees in their recovery from the psychological impact of exposure to persecution, conflict, and displacement.

In recent years, considerable research attention has been devoted to identifying factors that contribute to the gap between mental health needs and treatment uptake in refugees. Such factors associated with reluctance to engage in help-seeking can include both structural and cultural barriers. Specifically, language proficiency, financial strain, difficulties accessing transport, lack of knowledge about available mental health services, insecure immigration status, resettlement living difficulties, insecure housing, and mental health stigma have all been associated with reduced help-seeking in refugees (Boettcher et al., 2021; Byrow et al., 2019, 2020; Tomasi et al., 2022). In contrast, relatively less research attention has been paid to negative beliefs regarding the help-seeking process (negative help-seeking beliefs). This is despite evidence that such beliefs are highly prevalent in refugee communities and that they contribute to lower treatment uptake in refugees and other groups, including military personnel, young people, and the general population (Byrow et al., 2020; Gulliver et al., 2010; Schlechter et al., 2021; ten Have et al., 2010; Vogt, 2011).

Help-seeking beliefs are likely to present differently in refugees compared with other groups given the unique refugee experience. First, exposure to interpersonal trauma and forced displacement may give rise to common themes of beliefs about help-seeking that impact on treatment access for refugees. Second, help-seeking beliefs are likely influenced by cultural norms and values. As such, in our investigation of the literature regarding these beliefs, we focused on studies undertaken with refugees from collectivist backgrounds as (a) these represent a large proportion of forcibly displaced persons in the contemporary context (United Nations High Commissioner for Refugees [UNHCR], 2023) and (b) collectivism represents an important cultural dimension likely to influence help-seeking beliefs (Mojaverian et al., 2013). Our literature search indicated that negative help-seeking beliefs in refugees can be broadly categorized into four key themes.

The first theme focuses on fears relating to seeking treatment. The available research evidence suggests that these fears can take several forms. For example, refugees may fear that personal matters shared in the context of therapy will be communicated to others in their community, especially when a mental health practitioner or interpreter shares the same ethnic, cultural, or religious background as the client (Colucci et al., 2015; de Anstiss & Ziaian, 2010; Shannon et al., 2015; Soukenik et al., 2022). These confidentiality fears may culminate in the perception that treatment has potential negative consequences (Slewa-Younan, Radulovic, et al., 2014). These fears may be heightened by beliefs that talking to a mental health professional about past traumatic experiences may cause a loss of emotional control, exacerbate emotional problems, and/or lead to hospitalization or separation from family (Bettmann et al., 2015; Dinos et al., 2004; Shannon et al., 2015; Tran et al., 2021). In addition, refugees may fear that disclosing information in the context of therapy will risk the outcome of their visa applications or the safety of family still living in the country of origin, for example, if information disclosed in therapy is shared with government officials in the host or home country (Bettmann et al., 2015; de Anstiss & Ziaian, 2010; Piwowarczyk et al., 2014; Shannon et al., 2015).

The second theme relates to the belief that discussing mental health difficulties with a stranger (even if they are a mental health professional) is unhelpful or inappropriate (Bettmann et al., 2015; Piwowarczyk et al., 2014). Accordingly, individuals may have beliefs that psychological treatment is ineffective, or even that seeking psychological treatment is transgressing important social or cultural norms. These beliefs may be magnified when refugee clients work with mental health practitioners with cultural backgrounds that are different to their own. This cultural discrepancy can give rise to concerns that these professionals will not be able to fully understand the individual’s experience or will judge him or her negatively (de Anstiss & Ziaian, 2010; Drummond et al., 2011; Shannon et al., 2015; Soukenik et al., 2022; Tran et al., 2021).

The belief that individuals can or should cope with mental health difficulties on their own has also been commonly reported in refugees (Slewa-Younan, Mond, et al., 2014; Tobin et al., 2018). This may be reflected in the perception that help-seeking is only reserved for severe mental health difficulties and is, therefore, unnecessary especially when informal sources of help may be preferred (Grupp et al., 2019; Renner et al., 2020; Tobin et al., 2018). Consistent with this, men who subscribe to dominant masculine values, including stoicism, have been found to hold more negative beliefs about help-seeking (Vogel et al., 2011). These beliefs may be especially prevalent in cultures where gender norms are clearly defined. Accordingly, a study with male Syrian refugees found that self-reliance and refusing help were perceived as integral components of dignity, with the concept of dignity being highly valued in Syrian culture and intrinsic to identity (Grandi et al., 2018; Wells et al., 2018). Therefore, a strong cultural emphasis on stoicism may lead formal psychological support to be perceived as a direct threat to self-esteem, identity, and cultural values.

The fourth theme relates to mental health stigma, which research has demonstrated is prevalent in refugee communities (Kira et al., 2014; Satinsky et al., 2019). Mental health stigma is an overarching term that encompasses negative stereotypes related to symptoms and help-seeking (Corrigan et al., 2014). Stigmatizing beliefs can exist at the community level resulting in prejudice and discrimination (public stigma) and individual level whereby stigmatizing beliefs endorsed by the community are internalized to the self (self-stigma; Corrigan, 2000; Vogel et al., 2007; Watson et al., 2007). Stigmatizing beliefs related to mental health and help-seeking are well-documented barriers to accessing support in their own right among refugee communities and various other groups (Clement et al., 2015; Satinsky et al., 2019). One way by which stigma may lead to reduced help-seeking is when an individual considers seeking mental health support from a health professional to be shameful and fears that it may lead to negative judgment from others in the community toward themselves and their family (Kiselev et al., 2020; Slobodin et al., 2018; Valibhoy et al., 2017). As such, the perception of negative social consequences associated with help-seeking, including ostracism and/or jeopardizing marriage prospects, is cited as a prominent help-seeking deterrent (de Anstiss & Ziaian, 2010; Shannon et al., 2015; Yassin et al., 2018).

There is strong evidence that negative beliefs about help-seeking in refugees are related to reduced help-seeking behaviors. However, while scales measuring stigma and general help-seeking beliefs and attitudes exist, there is currently no scale that has collated and systematically measured negative beliefs regarding help-seeking as they specifically relate to refugees. This is problematic as, given the nature of the refugee experience (exposure to interpersonal trauma in the context of persecution and war leading to potential distrust of health care professionals, displacement to a new environment with unfamiliar health care structures), refugees are likely to report distinct negative beliefs that impact on help-seeking. Other measures of help-seeking beliefs that have been used cross-culturally are either limited to measuring the perceived efficacy of various interventions or focus on beliefs about symptom etiology and subsequent treatment options (Al-Krenawi et al., 2009; Schlechter et al., 2021; Slewa-Younan, Mond, et al., 2014). This presents a gap in the literature given that negative help-seeking beliefs are a key barrier to seeking formal support in traumatized refugees. The development of a measure of negative help-seeking beliefs specifically designed for refugees would provide critical information for increasing treatment uptake in a population with high mental health needs and low help-seeking.

In this study, we drew on existing qualitative and quantitative research to develop and psychometrically validate a novel measure assessing negative beliefs about help-seeking in refugees. We developed this measure for individuals from a refugee background, paying particular attention to assessing constructs that are relevant in collectivist cultures. As such, this initial study was conducted with a sample of Arabic-speaking refugees in recognition that a large proportion of the world’s refugees are forcibly displaced from Arabic-speaking countries (UNHCR, 2023). We aimed to identify the factor structure and assess the internal consistency and convergent validity of the novel measure. Based on the literature, it was hypothesized that items would load onto four factors capturing different types of help-seeking beliefs: (a) Fear of Negative Consequences, (b) Inappropriateness, (c) Perceived Necessity, and (d) Social Repercussions. Regarding convergent validity, it was hypothesized that negative help-seeking beliefs would be positively associated with related constructs such as domains of stigma (self-stigma related to PTSD, self-stigma related to help-seeking and perceived stigma) and negative help-seeking attitudes. In addition, it was hypothesized that negative help-seeking beliefs would be associated with lower help-seeking intentions for refugees with high levels of PTSD symptoms. We report how we determined our sample size, all data exclusions, all manipulations, and all measures in the study.

## Materials and Methods

### Participants

Participants were 262 refugees living in Australia who met the following inclusion criteria: (a) refugee or asylum-seeker background, (b) literate (i.e., able to read and write) in Arabic, and (c) over 18 years of age. Participants in this study were recruited from advertising at refugee centers and services, social media platforms (i.e., Facebook), and snowball sampling where participants were asked to indicate whether someone they knew would be interested in participating. Snowball sampling has been found to be an effective recruitment method to reach difficult-to-access populations such as refugees (Sadler et al., 2010).

### Measures

All measures were translated into Arabic, and then blind back-translated into English by accredited translators. Minor discrepancies were reconciled in collaboration with Arabic translators experienced in working with mental health–related material.

#### Development of the Help-Seeking Beliefs Scale

A 30-item scale was developed to assess common beliefs that are associated with reduced help-seeking for mental health problems in refugees. Items were initially generated by the study authors following a literature search on this topic. Following a co-design participatory approach, these items were reviewed and refined in consultation with (a) clinical psychologists experienced in providing psychological treatment to Arabic-speaking refugees and (b) Arabic-speaking individuals from a refugee background who were familiar with mental health–related content. Following this process, items were amended for appropriateness, relevance, expression, and clarity. The scale included items relating to the broad themes identified in the literature associated with help-seeking: (a) Fear of Negative Consequences, (b) Inappropriateness, (c) Perceived Necessity, and (d) Social Repercussions. Participants respond on a 4-point scale indicating the extent to which they agree or disagree with each belief (1 = *strongly disagree* to 4 = *strongly agree*). Higher ratings indicate greater negative help-seeking beliefs.

#### Exposure to Potentially Traumatic Events

The 16-item Harvard Trauma Questionnaire (Mollica et al., 1992) was used to assess exposure to potentially traumatic events (PTEs) commonly experienced by refugees (e.g., lack of food or water, serious injury, murder of family or friends) and is widely used among populations that have experienced mass conflict and displacement (Mollica et al., 1998; Sigvardsdotter et al., 2016; Steel et al., 2009). An additional 4 items were included to measure other PTEs refugees may experience; “Family Violence”; “Serious accident at work, home, or during recreational activity”; “Physical assault”; “Assault with a weapon”. Participants indicate whether they have experienced, witnessed, or learnt about each event or none of the above. In this study, endorsing “experienced,”“witnessed,” or “learnt about” was considered to constitute an affirmative response. Positive responses were summed to create a count indexing exposure to PTEs ranging from 0 to 20.

#### Posttraumatic Stress Symptoms

The 20-item Posttraumatic Diagnostic Scale–5 (PDS-5; Foa et al., 2016) was used to index PTSD symptoms. The PDS has been validated in Arabic and used in refugee samples (Alghamdi & Hunt, 2019; Bogic et al., 2015; Schock et al., 2016; Selmo et al., 2019). Items comprise PTSD symptoms in the *Diagnostic and Statistical Manual of Mental Disorders* (5th ed.; *DSM-5*; American Psychiatric Association, 2013) grouped under each cluster: re-experiencing, avoidance, negative alterations in mood and cognition and hyperarousal. Participants indicate how much each symptom has bothered them in the past month using a 5-point rating scale (0 = *not at all* to 4 = *6 or more times a week/severe*). A mean across all items was used to index PTSD symptom severity. Internal consistency for this scale in this study was α = .97.

#### Self-Stigma of PTSD

A 24-item version of the internalized stigma of mental illness scale (ISMI) was used to measure self-stigma of PTSD (SSPTSD; Ritsher et al., 2003). The ISMI is a highly sensitive and widely used measure in stigma research and with Arabic-speaking refugees (Kira et al., 2014, 2015; Mittal et al., 2012). Participants indicated the extent to which they agree with items assessing self-stigma in relation to symptoms of posttraumatic stress experienced (e.g., “I am embarrassed or ashamed that I have symptoms of posttraumatic stress”). The scale assesses different domains of self-stigma, including alienation, stereotype endorsement, discrimination experience, and social withdrawal. The original scale includes a stigma resistance subscale (e.g., “I feel comfortable being seen in public with an obviously mentally ill person”); however, this subscale has been found to reduce the internal consistency of the measure in previous studies and so was not included in the present study (Ritsher et al., 2003; Wastler et al., 2020). Participants respond to each self-stigma belief on a 4-point scale (1 = *strongly disagree* to 4 = *strongly agree*). A mean across all items was calculated with higher total scores indicating greater self-stigma related to PTSD. Internal consistency for this scale in this study was α = .98.

#### Self-Stigma of Seeking Help

A five-item version of the self-stigma of seeking help measure (SSOSH; Vogel et al., 2006) was used to assess self-stigma arising from seeking professional psychological help. The scale has strong psychometric properties and has been used with refugee samples (Byrow et al., 2019; Nickerson et al., 2019; Vogel et al., 2006). Participants indicated how they would feel if they sought professional help. Participants endorsed the extent to which they agree with each belief using a 5-point scale (1 = *strongly disagree* to 5 =*strongly agree*). The full 10-item version of this scale includes five items worded in the negative direction (e.g., “I would feel inadequate if I went to a therapist for psychological help”) and five items measured in the opposite direction (e.g., “My view of myself would not change just because I made the choice to see a therapist”). Upon examining psychometric properties of this measure, we found that negative items showed strong internal consistency (α = .85), as did positive items (α = .85), but (a) the two sets of items did not correlate strongly with one another and (b) the valence of the relationship (positive) was in the opposite direction than was expected (*r* = .36). Given this, and other evidence that items which need to be reverse-scored demonstrate poor internal consistency with the other items in cross-cultural research (Nickerson et al., 2015; Schlechter et al., 2021), we retained only the negatively worded items for analyses in this study. Internal consistency for this five-item version of the SSOSH scale was α = .85.

#### Perceived Stigma

The nine-item perceived stigma subscale of the depression stigma scale was included to capture perceived stigma (DSS; Griffiths et al., 2004). We adapted items to reference PTSD rather than depression. The scale assessed beliefs about the negative views held by others in relation to PTSD (e.g., “most people believe that people with symptoms of posttraumatic stress could snap out of it if they wanted”). We also added an additional item assessing the participants' perception of how a family (not just an individual) may be judged, to appropriately capture interdependent relationships in collectivest cultures (“most people would judge a family negatively if someone in that family had symptoms of posttraumatic stress”). The DSS has been used cross-culturally and is a sensitive, widely used measure of perceived stigma (Kiropoulos et al., 2011; Mittal et al., 2012). Participants responded on a 5-point scale (0 = *strongly disagree* to 4 =*strongly agree*). Mean scores were calculated whereby higher scores indicate greater levels of perceived stigma. Internal consistency for this scale in this study was α = .93.

#### Help-Seeking Attitudes

A five-item version of the attitudes toward seeking professional psychological help (short form) was used to assess help-seeking attitudes (ATSPPH-SF; Fischer & Farina, 1995). Items in this study assessed attitudes against seeking professional psychological help (e.g., “Considering the time and expense involved in psychotherapy, it would have doubtful value for a person like me”). These five items had strong internal consistency (α = .88) as did items in the opposite direction from the full 10-item scale (e.g., “If I believed I was having a mental breakdown, my first inclination would be to get professional attention”; α = .90). However, similar to the SSOSH, both sets of items did not correlate strongly and had a positive relationship despite being worded in opposite directions (*r* = .33), a similar finding to another study that included a refugee sample (Schlechter et al., 2021). Therefore, only items worded in the negative direction were retained for analyses. Participants responded on a 4-point scale (0 = *disagree* to 3 = *agree*). Internal consistency for this five-item version of the ATSPPH scale was α = .88.

#### Help-Seeking Intentions

An adapted version of the General Help-Seeking Questionnaire (Wilson et al., 2005) was used to measure the likelihood of participants seeking help in the next 4 weeks from 12 potential help-seeking sources. Help-seeking sources from the original measure were adapted to better reflect potential sources of help within the Australian context, and those available for refugee clients (e.g., “social worker” was replaced with “caseworker”) consistent with other refugee studies that have used this scale (Byrow et al., 2019; Nickerson et al., 2019). The help-seeking sources assessed in this study were either formal (e.g., mental health professionals such as a psychologist, health professionals such as a doctor, or other professionals such as a caseworker) or informal (e.g., friend, family member). Participants also had the option to indicate the likelihood they would not seek help. Participants responded on a 7-point scale (1 = *extremely unlikely*, 3 = *unlikely*, 5 = *likely*, 7 = *extremely likely*). The mean ratings averaged across all 12 sources reflected general help-seeking intentions with higher scores indicating greater intentions. Internal consistency for this scale in this study was α = .90.

### Procedure

Data collection was undertaken between November 2020 and December 2020. Potential participants were invited to take part in the online survey via a Qualtrics link. Participants who were interested in taking part clicked on the link and completed online informed consent procedures before completing the online survey (consisting of the questionnaires above). Of the potential participants invited, 39% completed the survey. The survey took approximately 45 minutes to 1 hour to complete. Upon completion, participants were reimbursed with a $AUD20 online voucher to compensate them for costs undertaking the study. All procedures involving participants were approved by the UNSW Human Research Ethics Committee, HC200709.

### Data Analysis

A series of analyses were conducted to determine the reliability and validity of the Help-Seeking Beliefs Scale (HSBS) as well as the factor structure and items to retain. We then examined the association of the HSBS with other key related measures to determine convergent validity.

To assess the factor structure of the HSBS, the total sample (*n* = 262) was randomly split into subsamples (using the SPSS function). An exploratory factor analysis (EFA) was conducted on the first sample (*n* = 150) to investigate the underlying factor structure and to reduce the item pool. Confirmatory factor analysis (CFA) was conducted on the second sample (*n* = 112) to provide initial validation of the factor structure. A larger sample was used for the EFA given the item pool was larger (150 participants for 30 items, allowing for a 5:1 subject to item ratio) and a smaller sample for the CFA with the reduced item set (facilitating a minimum of 5:1 subject to item ratio) (Worthington & Whittaker, 2006).

EFA analyses were conducted using SPSS version 26. The missing data rate was 7.3% and was missing completely at random (MCAR; *p* = .993); therefore, listwise deletion was applied for missing items, which is considered unbiased when data meet the MCAR assumption and have a missing data rate of less than 10% (Centre for Behavioral Health Statistics and Quality, 2018; Kang, 2013). Prior to performing the EFA, data were assessed for factor analysis suitability as indicated by a value of 0.6 or greater in the Kasier–Meyer–Olkin (KMO) Measure of Sampling Adequacy test and a significant *p* value (*p* > .05) in Bartlett’s Test of Sphericity (Tabachnick & Fidell, 2007). Kaiser’s criterion (retaining factors only with eigenvalues greater than one; Kaiser, 1960) and parallel analysis (Horn, 1965) were used to determine the number of factors to retain. Parallel analysis was conducted using syntax for principal axis factoring to generate 1,000 random data sets based on sample size and number of items (O’Connor, 2000). Factors retained had eigenvalues larger than the 95th percentile eigenvalue of the corresponding factor in the random data sets (O’Connor, 2000). Factors were rotated using oblique rotation (Promax method) given that factors were expected to correlate (Tabachnick & Fidell, 2001). Item retention was theoretically and psychometrically driven by examining the pattern matrix factor loadings and the correlation matrix (Costello & Osborne, 2005). The following criteria for item removal were implemented: (a) items with factor loadings below 0.40 (Hair et al., 2010), (b) collinearity (>0.8) (Tabachnick & Fidell, 2013), (c) cross-loading on more than one factor (.40) (Hair et al., 2014), and (d) items that loaded onto theoretically dissimilar items.

The factor solution of the reduced scale was tested in the remaining participants (*n* = 112) using CFA. In addition to verifying the indicator items with their respective factors, utilizing a second-order model also allowed us to test the extent to which the three first-order factors represented the HSB construct. Specifically, a second-order model was tested with three first-order factors (Fear of Negative Consequences, Inappropriateness, Perceived Necessity) as indicators of an overarching second-order factor (HSB). This model was compared with a single-factor structure to determine whether the three factors demonstrated the best fit to the data. Analyses were conducted using Mplus version 8.8 (Muthén & Muthén, 1998–2022) using a maximum likelihood estimator (MLR) recommended for small to medium sample sizes (Wang & Wang, 2012). There was less than 5% missing data. Model fit was assessed using the following criteria: the root mean square error of approximation (RMSEA) close to 0.06 or lower, comparative fit index (CFI) and the Tucker–Lewis index (TLI) close to 0.95 or higher, and standardized root mean square residual (SRMR) close to 0.08 or lower (Hu & Bentler, 1999).

After factor analyses, the following analyses were conducted on the total sample to increase power (*n* = 262). To establish internal consistency, McDonald’s Omega (ω) was reported alongside Cronbach’s alpha (α) given that the former is a more robust measure of reliability (Goodboy & Martin, 2020; McNeish, 2018; Sijtsma, 2009). Coefficient values > 0.7 represent good internal consistency (McNeish, 2018). To examine convergent validity, Pearson’s correlations investigated whether the total HSBS mean score and subscale means were correlated with similar constructs (i.e., self-stigma of PTSD, self-stigma of help-seeking, perceived stigma and help-seeking attitudes). Constructs were expected to be significantly related in the positive direction.

When investigating whether HSBS scores predicted help-seeking, we also considered PTSD symptom severity, given that individuals with low levels of PTSD symptoms would be unlikely to seek psychological treatment. As such, we examined whether the association between HSBS scores and help-seeking differed according to PTSD symptom severity. Specifically, we used moderated multiple regression analyses to examine the association between HSBS, PTSD, and their interaction and help-seeking. Continuous predictors (HSBS and PTSD) were grand mean-centered and the interaction term (PTSD × HSB) was calculated from the centered values. Both predictors were entered at the first step and the interaction variable was entered at the second step. Simple slopes analyses were used to delineate significant interaction effects for participants with high (+1 *SD*) and low (–1 *SD*) PTSD symptoms.

## Results

### Participant Characteristics

Among the total sample (*n* = 262), participants had a mean age of 42.79 (*SD* = 13.30) years and just over half of the sample were female (*n* = 133, 50.8%). See Table 1 for detailed participant characteristics.

**Table 1:** Participant Characteristics.

Demographics	*N* (%)
Age	*M* = 42.79; *SD* = 13.30
Gender (female)	133 (50.8)
Country of birth	
Iraq	213 (81.3)
Syria	40 (15.3)
Egypt	4 (1.5)
Other	5 (1.9)
Relationship status	
Married or in a relationship	202 (77.1)
Not married or in a relationship	60 (22.9)
Education	
Little or no formal education	9 (3.4)
Completed primary school (age 11)	28 (10.7)
Completed high school (age 16/18)	71 (27.1)
Completed university	118 (45.0)
Completed other training (vocational, apprenticeship)	36 (13.7)
Time in Australia (years)	*M* = 4.31; *SD* = 1.74
Trauma exposure (PTEs experienced)	*M* = 6.57; *SD* = 7.15

*Note.* PTEs = potentially traumatic events.

### Factorial Validity

Principal axis factor analysis was conducted on the initial 30-item pool in the EFA sample (*n* = 150). The KMO value (0.91) and the Bartlett test (*p* < .001) indicated that the data were highly suitable for factor analysis. The initial factor analysis revealed four factors with eigenvalues greater than one after extraction (and before rotation) supported by parallel analysis. The four-factor solution explained 67.04% of the variance (Factor 1: 47.56%, Factor 2: 10.35%; Factor 3: 5.47%; Factor 4: 3.67%). Another EFA (principal axis factoring) was conducted on a fixed number of four factors applying oblique (Promax) rotation to aid in interpretation. The fixed four-factor solution explained 65.89% of the variance (Factor 1: 47.28%, Factor 2: 10.05%; Factor 3: 5.18%; Factor 4: 3.38%).

Next, the pattern matrix and correlation matrix were examined to determine which items to retain. Three items cross loaded (>0.40) on two factors and were therefore removed (“Emotional problems are best dealt with inside the family rather than seeing a mental health professional”; “There are certain problems which should not be discussed outside of one’s immediate family”; “If I spoke to a mental health professional about my emotional problems, I would worry that they would tell others in my community about it”). No item had a factor loading < 0.40 and there was no evidence of singularity. There were, however, three pairs of collinear items (i.e., correlations > .8), with one item included in two of the three pairs. All of these pairs loaded onto Factor 4 and were phrased in the same way referring to the individual (e.g., “If I saw a mental health professional my community would judge me negatively”) or their family (e.g., “If I saw a mental health professional my community would judge my family negatively”). Given the high collinearity within the factor, items referring to community judgment about the individual (rather than the family) were retained to ensure consistency within the factor.

Altogether five items were removed and the fixed four-factor solution was again examined applying oblique (Promax) rotation. With the items removed, parallel analysis and eigenvalues suggested a three-factor solution. As such, a fixed three-factor solution was examined applying oblique (Promax) rotation. Only one item, “People should be able to cope with emotional problems without seeing a mental health professional” cross loaded onto two factors and was subsequently removed. There was no evidence to support the removal of any other items. The three-factor solution was examined again with oblique (Promax) rotation following the removal of the item. Parallel analysis and eigenvalues still supported the three-factor solution and there was no evidence to support the removal of any other items.

Therefore, the final solution contained 24 items and explained 62.14% of variance (see factor loadings in Table 2 and items in Table 3). Factor 1, which we labeled “Fear of Negative Consequences,” comprised 11 items relating to perceived negative consequences associated with mental health treatment, including fears of confidentiality breaches, exacerbation of symptoms, and discrimination from the community. This factor explained 47.11% of the variance. Factor 2 or “Inappropriateness” comprised eight items relating to discomfort about disclosing private matters to mental health professionals and perceived lack of cultural competency. This factor explained 9.55% of the variance. Finally, Factor 3 or “Perceived Necessity” comprised five items relating to stoic attitudes rendering mental health professionals unnecessary. This factor explained 5.49% of the variance.

**Table 2:** Factor Structure Loadings for the Help-Seeking Beliefs Scale From the EFA and CFA.

Items	EFA sample (*n* =150)			CFA sample (*n* = 112)		
	Factor 1	Factor 2	Factor 3	Factor 1	Factor 2	Factor 3
HSB1	**0.807**	0.025	−0.015	0.80		
HSB2	**0.718**	0.185	−0.079	0.86		
HSB3	**0.905**	−0.032	−0.127	0.84		
HSB4	**0.848**	−0.022	−0.075	0.87		
HSB5	**0.743**	0.113	0.056	0.92		
HSB6	**0.860**	−0.093	0.046	0.90		
HSB7	**0.817**	−0.086	0.120	0.90		
HSB8	**0.711**	−0.030	0.164	0.79		
HSB9	**0.691**	0.051	0.114	0.83		
HSB10	**0.600**	0.076	0.133	0.83		
HSB11	**0.659**	0.137	0.103	0.88		
HSB12	−0.143	**0.783**	0.050		0.80	
HSB13	0.016	**0.720**	0.079		0.83	
HSB14	−0.042	**0.827**	0.065		0.86	
HSB15	−0.089	**0.763**	0.125		0.87	
HSB16	0.014	**0.593**	0.139		0.86	
HSB17	0.192	**0.725**	−0.098		0.84	
HSB18	0.302	**0.743**	−0.194		0.82	
HSB19	0.134	**0.687**	0.078		0.84	
HSB20	0.152	0.083	**0.638**			0.84
HSB21	0.242	0.000	**0.626**			0.88
HSB22	0.184	0.038	**0.532**			0.81
HSB23	−0.111	0.047	**0.782**			0.85
HSB24	−0.023	0.066	**0.761**			0.86
HSB (second-order factor)				0.86	0.89	0.78

*Note.* Principal axis factoring (Promax Rotation) was used for factor extraction in the EFA analysis. Factor 1: Fear of Negative Consequences; Factor 2: Inappropriateness; Factor 3: Perceived Necessity. Boldfaced values indicate the highest factor loading. CFA factor loadings are standardized. EFA = exploratory factor analysis; CFA = confirmatory factor analysis; HSB = help-seeking beliefs.

**Table 3:** HSB Items.

Item number	Item
HSB1	If I spoke to a mental health professional about personal matters, I would worry that they might tell others in authority, such as government or immigration officials.
HSB2	Mental health professionals cannot be trusted
HSB3	Mental health professionals cannot be trusted to keep information private
HSB4	If I spoke to a mental health professional about emotional problems, I would worry that others in my community will find out about it
HSB5	Talking to a mental health professional about past experiences would make emotional problems worse
HSB6	Talking to a mental health professional could have negative consequences (e.g., being sent to hospital or being separated from family)
HSB7	Talking to a mental health professional about past experiences may cause me to lose control emotionally
HSB8	My family would not approve of me seeing a mental health professional as a way to deal with emotional problems
HSB9	If I saw a mental health professional, my community would judge me negatively
HSB10	Seeing a mental health professional could jeopardize marriage prospects for someone in my community
HSB11	If I saw a mental health professional, I might be ostracized by my community
HSB12	Emotional problems should be kept private from strangers even if they are mental health professionals
HSB13	I would feel uncomfortable discussing private matters with strangers including mental health professionals
HSB14	It is not appropriate to discuss private matters with strangers even if they are mental health professionals
HSB15	Mental health professionals would not understand the emotional problems experienced by people in my community
HSB16	Mental health professionals from a different culture would not understand the emotional problems I experience
HSB17	Mental health professionals would judge people from my community negatively if they talk to them about their emotional problems
HSB18	Mental health professionals would judge me negatively if I talk to them about my emotional problems
HSB19	People shouldn’t burden others with their emotional problems even if they are mental health professionals
HSB20	A person should be able to deal with their emotional problems alone
HSB21	Seeing a mental health professional is not necessary because other people can help with emotional problems (e.g., family members, spiritual leader)
HSB22	People should only see a mental health professional if they have very severe emotional problems
HSB23	My emotional problems are not severe enough to see a mental health professional
HSB24	Emotional problems tend to naturally improve without seeing a mental health professional

*Note.* HSB = help-seeking beliefs.

CFA was utilized to verify the three-factor solution with the reduced item set using the second split-half sample (*n* = 112). The initial second-order model demonstrated inadequate fit to the data (CFI = 0.880, TLI = 0.867, RMSEA = 0.093, SRMR = 0.062). As such, modification indices (MI) and theoretical considerations were used to guide model re-specification. Following this, we allowed two item pairs to covary. These two item pairs, “Mental health professionals would judge people from my community negatively if they talk to them about their emotional problems,”“Mental health professionals would judge me negatively if I talk to them about my emotional problems” and “Mental health professionals cannot be trusted,”“Mental health professionals cannot be trusted to keep information private” overlapped in content, wording, and themes, respectively. Therefore, these covariances were specified in the model, which yielded acceptable model fit (CFI = 0.917, TLI = 0.907, RMSEA = 0.078, SRMR = 0.058). As shown in Table 2, standardized regression coefficients ranged from 0.79 to 0.92 with all items loading significantly onto their respective factors. This model was compared with a single-factor model to ascertain whether the three factors were the best fit for the data. The single-factor solution had a poorer fit to the data compared with the second-order model with the three first-order factors. Fit statistics for both models are presented in Table 4.

**Table 4:** Model Fit Statistics for One-Factor and Three-Factor Higher Order Models.

Model	χ^2^	*df*	*p*	RMSEA	CFI	TLI	SRMR	AIC	SABIC
Single-factor	737.246	250	<.001	0.132	0.756	0.731	0.095	3,622.221	3,589.524
Three-factor	413.842	247	<.001	0.078	0.917	0.907	0.058	3,168.199	3,134.177

*Note.* RMSEA = Root mean square error of approximation; CFI = Comparative fit index; TLI = Tucker—Lewis index; SRMR = Standardized root mean square residual; AIC = Akaike information criterion; SABIC = Sample size adjusted BIC

### Reliability

With the final scale determined, reliability, convergent validity, and predictive validity were examined with the full sample (*n* = 262). Descriptive statistics are displayed in Table 5. Internal consistency for the final 24-item HSBS and its subscales were high, indicating good reliability (Total: α = .962, ω = 0.960; Fear of Negative Consequences: α = .958, ω = 0.958; Inappropriateness: α = .935, ω = 0.935; Perceived Necessity: α = .891, ω = 0.889). The internal consistency of either the full scale or subscales could not be improved with the deletion of items. Given that high Omega and Alpha coefficients in this study may reflect narrow measurement of the construct, we undertook corrected item-total correlations between the items and their subscales as well as the total scale to assess item convergence (Tables 6–9; Loevinger, 1954). We also investigated correlations between items and the nonparent subscales to assess item divergence (Tables 10–12). The corrected item-total correlations of the HSB items with the full scale ranged from 0.53 to 0.82 which suggests some variability in the scale while the items still converged on their respective subscales (0.67–0.86). The correlations between the nonparent subscales ranged from 0.37 to 0.64 indicating item divergence that is further supported by the absence of cross-loadings in the EFA analysis. The factors were also significantly related to one another (Inappropriateness and Fear of Negative Consequences: *r* = .66; Fear of Negative Consequences and Perceived Necessity: *r* = .60; Inappropriateness and Perceived Necessity: *r* = .61, all *p* values < .001).

**Table 5:** Descriptive Statistics for the HSBS and Subscales.

Factors	*M*	*SD*	Range	Skewness	Kurtosis
HSBS (total)	2.15	0.52	1.00–3.33	−0.37	0.08
Negative consequences (Factor 1)	2.01	0.57	1.00–3.45	−0.01	−0.34
Inappropriateness (Factor 2)	2.22	0.61	1.00–3.75	−0.21	−0.00
Perceived Necessity (Factor 3)	2.36	0.61	1.00–4.00	−0.54	0.03

*Note.* HSBS = Help-Seeking Beliefs Scale.

**Table 6:** HSB Items Corrected Item-Total Correlations for Full Scale

Item	Correct item-total correlations
HSB1	.63
HSB2	.70
HSB3	.73
HSB4	.68
HSB5	.67
HSB6	.70
HSB7	.73
HSB8	.73
HSB9	.72
HSB10	.75
HSB11	.70
HSB12	.73
HSB13	.82
HSB14	.75
HSB15	.76
HSB16	.72
HSB17	.75
HSB18	.73
HSB19	.79
HSB20	.70
HSB21	.68
HSB22	.63
HSB23	.53
HSB24	.61

*Note.* HSB = help-seeking beliefs.

**Table 7:** Factor 1 Items Corrected Item-Total Correlations for Factor 1 Subscale.

Item	Correct item-total correlations
HSB1	.78
HSB2	.80
HSB3	.80
HSB4	.81
HSB5	.86
HSB6	.84
HSB7	.85
HSB8	.76
HSB9	.79
HSB10	.76
HSB11	.82

*Note.* HSB = help-seeking beliefs.

**Table 8:** Factor 2 Items Corrected Item-Total Correlations for Factor 2 Subscale.

Item	Correct item-total correlations
HSB12	.73
HSB13	.76
HSB14	.82
HSB15	.80
HSB16	.74
HSB17	.78
HSB18	.78
HSB19	.80

*Note.* HSB = help-seeking beliefs.

**Table 9:** Factor 3 Items Corrected Item-Total Correlations for Factor 3 Subscale.

Item	Correct item-total correlations
HSB20	.74
HSB21	.77
HSB22	.67
HSB23	.74
HSB24	.76

*Note.* HSB = help-seeking beliefs.

**Table 10:** Correlations for Factor 1 Items With Nonparent Subscales.

Items	Factor 2	Factor 3
HSB1	.56**	.43**
HSB2	.59**	.47**
HSB3	.51**	.42**
HSB4	.55**	.44**
HSB5	.64**	.58**
HSB6	.53**	.51**
HSB7	.53**	.50**
HSB8	.51**	.55**
HSB9	.55**	.55**
HSB10	.56**	.52**
HSB11	.62**	.55**

*Note.* HSB = help-seeking beliefs.

** *p* < .001.

**Table 11:** Correlations for Factor 2 Items With Nonparent Subscales.

Items	Factor 1	Factor 3
HSB12	.48**	.46**
HSB13	.56**	.53**
HSB14	.56**	.53**
HSB15	.49**	.53**
HSB16	.51**	.54**
HSB17	.58**	.45**
HSB18	.63**	.46**
HSB19	.59**	.50**

*Note.* HSB = help-seeking beliefs.

** *p* < .001.

**Table 12:** Correlations for Factor 3 Items With Nonparent Subscales.

Items	Factor 1	Factor 2
HSB20	.59**	.56**
HSB21	.57**	.51**
HSB22	.53**	.51**
HSB23	.37**	.49**
HSB24	.45**	.52**

*Note.* HSB = help-seeking beliefs.

** *p* < .001.

### Convergent Validity

Pearson’s correlations revealed that the total HSBS and the three subscales significantly correlated with similar constructs in the expected direction and in the moderate range providing support for its convergent validity. A correlation matrix is presented in Table 8. Briefly, the mean score of the HSBS was positively correlated with self-stigma related to PTSD (*r* = .45, *p* < .001), self-stigma related to seeking help (*r* = .71, *p* < .001), perceived stigma (*r* = .66, *p* < .001), and negative attitudes related to help-seeking (*r* = .50, *p* < .001). These relationships suggest that the HSBS measure of negative help-seeking beliefs is significantly associated with other barriers toward help-seeking in refugees.

**Table 13:** Correlation Matrix of the HSBS, Subscales, and Validity Instrument Means.

Measures	1	2	3	4	5	6	7
(1) HSBS total							
(2) Neg consequences	.92**						
(3) Inappropriateness	.87**	.66*					
(4) Perceived necessity	.79**	.61**	.61**				
(5) SSPTSD	.45**	.49**	.35**	.25**			
(6) SSOSH	.71**	.65**	.66**	.51**	.58**		
(7) Perceived stigma	.66**	.61**	.57**	.55**	.41**	.52**	
(8) ATSPPH	.50**	.41**	.44**	.49**	.21**	.35**	.42**

*Note.* HSBS = Help-Seeking Beliefs Scale. SSPTSD = self-stigma of posttraumatic stress disorder; SSOSH = self-stigma of seeking help; ATSPPH = attitudes toward seeking professional psychological help.

* *p* < .05. **p < .001.

### Predicting Help-Seeking Intentions

**Figure 1.**
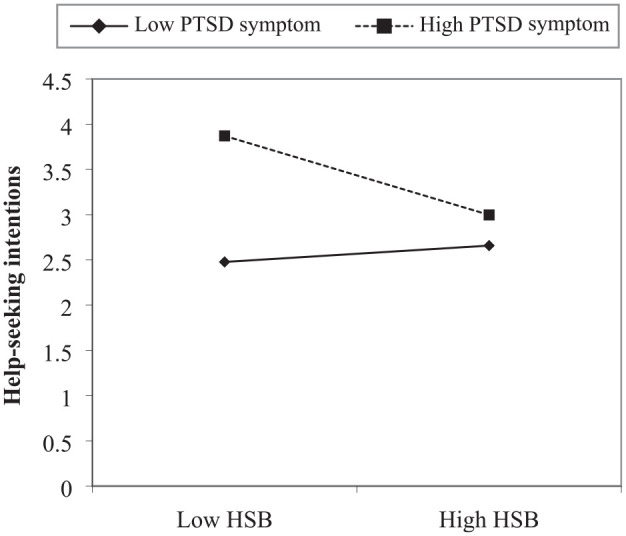
Simple Slopes Analysis of Interaction Between HSB and PTSD Symptoms Predicting Help-Seeking Intentions. *Note.* HSB = help-seeking beliefs; PTSD = posttraumatic stress disorder.

The results of moderated multiple regression analyses investigating the association between negative help-seeking beliefs, PTSD symptoms, and help-seeking intentions are presented in Table 9. PTSD symptoms significantly and positively predicted help-seeking intentions (β = 0.33, *p* < .001) while negative help-seeking beliefs significantly and negatively predicted help-seeking intentions (β = –0.14, *p =* .032). The association between the interaction of negative help-seeking beliefs and PTSD symptoms, and help-seeking intentions was significant (β = –0.21, *p* < .001). The results of simple slopes analyses at values one standard deviation above and below the mean are displayed in Figure 1. There was a significant negative association between negative help-seeking beliefs and help-seeking intentions for participants with higher PTSD symptoms (β = –0.85, *t* = −3.83, *p* < .001). Conversely, the association between help-seeking beliefs and help-seeking intentions was not significant for participants with lower PTSD symptoms.

**Table 14:** Moderated Multiple Regression Analyses Examining the Impact of Help-Seeking Beliefs and PTSD Symptoms on Help-Seeking Intentions.

	Help-seeking intentions			95% confidence intervals	
Model	*B* (*SE*)	β	*t*	Lower bound	Upper bound
Step 1 (*R*^2^ch)	0.11				
HSB	−0.34 (0.16)	−0.14	−2.15*	−0.64	−0.03
PTSD symptoms	0.59 (0.11)	0.33	6.00**	0.37	0.81
Step 2 (*R*^2^ch)	0.05				
HSB × PTSD symptoms	−0.69 (0.20)	−0.21	−3.49**	−1.08	−0.30

*Note.* HSB = Help-seeking beliefs; PTSD = posttraumatic stress disorder; *R*^2^ch = *R*^2^ change.

*p < .05. **p < .001.

## Discussion

The aim of the current study was to develop and psychometrically validate a novel measure of negative help-seeking beliefs in Arabic-speaking refugees. The measure was developed based on a literature search and consultation with clinical psychologists and professionals from a refugee background who were experienced in working with mental health material. An EFA on a subsample of participants revealed a three-factor structure. This was confirmed in a CFA on a separate subsample of participants. The utility of the three subscales is further supported by the three-factor model demonstrating better fit to the data compared with the single-factor model. Given that the CFA also confirmed the presence of a higher-order factor (HSB), this suggests the scale can be used flexibly; either as a total score to represent negative help-seeking beliefs, or the three subscale scores that represent subdomains of negative help-seeking beliefs. Findings are consistent with the theoretical understanding of negative help-seeking beliefs given that the three subscales map onto key themes of negative help-seeking beliefs identified in the literature: (a) Fear of Negative Consequences, (b) Inappropriateness, and (c) Perceived Necessity (Byrow et al., 2020).

Factor 1 “Fear of Negative Consequences” relates to fears about seeking treatment from a mental health professional, including confidentiality concerns, fear of exacerbation of symptoms, being hospitalized or separated from family, and ostracism from the community. Contrary to hypotheses, items related to social repercussions of seeking help loaded onto this factor rather than forming a distinct factor. This suggests that potential social consequences (such as community judgment and subsequent discrimination) are related to other anticipated, negative consequences that participants fear should they seek help. Indeed, ostracism from the community may be perceived as particularly detrimental in collectivist cultures where self-identity is defined based on interdependent social relationships and connectedness with the ingroup (Brewer & Chen, 2007).

Generally, items in this factor capture an underlying sentiment of distrust of mental health professionals (i.e., breaching confidentiality and making symptoms worse) which is likely to arise due to the nature of the refugee experience. Specifically, refugees often experience gross human rights violations perpetrated by authority figures or other people in the context of war and persecution. These experiences may contribute to reduced trust in other people, including professionals in the host country, and a reluctance to disclose and trust strangers with personal matters (Beltran et al., 2008; Colucci et al., 2015; Nickerson et al., 2014; Soukenik et al., 2022). Other perceived negative consequences may arise due to limited knowledge about treatment processes in the Western context (e.g., the requirement for information to be confidential). This may be exacerbated when the individual comes from a context where mental health treatment is less common due to limited funding, stigma, shortage of professionals, and reliance on traditional and/or religious methods (Al-Krenawi, 2005; Yehia et al., 2014). The finding that fears relating to help-seeking represented a central component of negative help-seeking beliefs in refugees suggests that addressing these fears (e.g., by providing clear information regarding confidentiality and consequences of help-seeking) may be an important strategy for increasing treatment uptake in refugees.

Factor 2, “Inappropriateness” relates to the discomfort associated with disclosing difficulties to mental health professionals. Items in this factor include beliefs regarding perceived low cultural competency of the professional and preference to keep mental health problems within the family. Refugees may be from cultural backgrounds that are different to that of the treating professional; this may give rise to a cultural disconnect between the client and services in the host country (Soukenik et al., 2022). This disconnect may lead to concerns as to whether the professional can adequately understand the client’s difficulties as well as the unique problems faced by the community and therefore appropriately meet their needs (de Anstiss & Ziaian, 2010). The preference to seek help from informal sources of support (such as immediate family) is well-documented among refugees and was originally conceptualized in this study as contributing to Factor 3 or lack of necessity of mental health services (Byrow et al., 2020). However, these items unexpectedly loaded onto the factor relating to inappropriateness of mental health treatment, which may be because seeking help from family is preferred to seeking help from a stranger, such as a mental health professional (Kiselev et al., 2020; Piwowarczyk et al., 2014). This is likely related to an emphasis on privacy about mental health difficulties due to mental health stigma in the community and aligns with collectivist cultural values (Kiselev et al., 2020; Kuo, 2013). Despite the preference for informal supports, Aarethun and colleagues (2021) found this to be context-specific whereby participants preferred to seek support from their social network in Syria, whereas in the host country, seeking formal support was considered to be more feasible as social networks are not fully established and mental health stigma may be less common among citizens of the host country. This finding highlights the importance of enhancing cultural competency among mental health professionals working with refugees. Furthermore, these results underscore the central role of refugee community members in providing mental health support. This is consistent with a growing body of literature supporting task-shifting approaches whereby lay individuals or paraprofessionals from within refugee communities provide mental health interventions (Galvin & Byansi, 2020). Future research should investigate whether beliefs about the appropriateness of formal mental health treatment interact with the type of treatment provider (e.g., mental health professional from outside community, paraprofessional from within community) to impact on help-seeking willingness and treatment efficacy.

Factor 3 “Perceived Necessity” relates to beliefs that one should cope on their own rendering mental health treatment unnecessary. As previously discussed, a strong cultural value of coping with difficulties is to be self-reliant, which is in direct conflict with seeking help (Wells et al., 2018). This may be because the needs of the collective are prioritized rather than the individual (Anderson & Losin, 2017; Papadopoulos et al., 2013). The literature suggests that both men and women with a refugee background endorse stoic beliefs but for different reasons; for men, upholding pride and notions of masculinity may be considered centrally important, while for women, focusing on their family’s needs (rather than prioritizing individual needs) and avoiding stigmatization particularly related to sexual violence and postpartum depression may lead to a stoic attitude to coping (Slobodin et al., 2018; Tobin et al., 2018; Vogel et al., 2011; Yohani & Okeke-Ihejirika, 2018). While the sample size precluded gender comparisons in the current study, future research should explore which help-seeking beliefs are more prominent for each gender. Nevertheless, preserving self-esteem and identity, regardless of gender, may be particularly important for refugees. This is likely to be the case as, in addition to trauma, displacement from the home country already means leaving behind parts of one’s identity (education, profession, social status) only to become one of many forcibly displaced people resettled in a new country (Slobodin et al., 2018). This can exacerbate feelings of worthlessness and low self-esteem (Misra et al., 2006; Slobodin et al., 2018). Therefore, in this resettlement context, upholding valued parts of identity and culture may be especially important even if it comes in direct conflict with recovery. As such, future research could explore how identity and self-esteem interact with beliefs about help-seeking (specifically stoic beliefs) to influence help-seeking behavior and whether this varies between type of help sort (formal vs. informal).

The final scale and the three subscales demonstrated excellent internal consistency suggesting that the HSBS is a reliable measure. Pearson’s correlations revealed that the scale correlated with similar constructs (self-stigma related to PTSD and help-seeking, perceived stigma and negative attitudes related to help-seeking) providing evidence for convergent validity. Previous research has shown these constructs to be key barriers to help-seeking in refugees (Byrow et al., 2019; Schlechter et al., 2021; Wachter et al., 2018). The moderate strength of the correlations provides support for the hypothesis that the construct of help-seeking beliefs is a related but distinct concept to attitudes and stigma. Future research could implement statistical techniques such as path analysis to examine the associations between all these concepts and help-seeking to identify the unique and distinctive roles of each.

Furthermore, moderated multiple regression analyses revealed that greater PTSD symptoms predicted greater help-seeking intentions while greater negative help-seeking beliefs predicted reduced intentions to seek help. The interaction effect was also significant; simple slopes analyses revealed that negative help-seeking beliefs are associated with reduced help-seeking intentions for participants with high PTSD symptoms, whereas this effect was not significant for participants with low PTSD symptoms. This may suggest that negative help-seeking beliefs can adversely impact intent to seek help for people who require mental health support the most. Given that higher PTSD symptoms predicted greater help-seeking intentions, the interaction findings may suggest that negative help-seeking beliefs interfere with the natural help-seeking trajectory if people experience symptoms. Overall, the results empirically demonstrate the association between negative help-seeking beliefs and reduced intentions to seek help consistent with qualitative studies, providing further support for the validity of the HSBS (Byrow et al., 2020).

It is also notable that, in this study, unidimensional scales (Self-Stigma of Seeking Help and Attitudes Toward Seeking Professional Psychological Help) that had been previously validated cross-culturally demonstrated poor internal consistency when considering items worded in conflicting directions for this sample (Rayan et al., 2020; Vogel et al., 2013). This has also been observed in other refugee studies where scales have been translated (Nickerson et al., 2015; Schlechter et al., 2021). Indeed, Schlechter and colleagues (2021) also observed this effect in the ATSPPH scale among Arabic-speaking refugees and Zhou and colleagues (2019) observed a similar effect in the SSOSH among Chinese students. This may be because reverse-worded items tend to reduce reliability and introduce an extra layer of complexity when comprehending and responding to questions particularly in cross-cultural research (Baumgartner & Weijters, 2015; Weijters et al., 2013). The present results add to the growing evidence cautioning the use of reverse-worded items when translating measures in cross-cultural research.

Findings from this study should be interpreted while taking into account limitations. First and foremost, the low response rate reduced the sample size that may have contributed to a less than optimal RMSEA (Kyriazos, 2018). Given refugees are a difficult-to-reach population, increasing the sample size proved challenging. As such, we aimed for a 5:1 subject to item ratio that was appropriate for the current sample given the significant Bartlett’s test of sphericity and the KMO value of 0.91 (above the recommended 0.6 cut-off; Worthington & Whittaker, 2006). Therefore, while the current study provides preliminary validation of the scale’s factor structure and psychometric properties, future studies with access to larger refugee samples should further validate the HSBS. The relatively small sample size also precluded ability to undertake further analyses such as gender comparisons. As alluded to previously, this is an important consideration given preferences for coping and help-seeking barriers can differ between men and women from refugee backgrounds (Renner & Salem, 2009).

Second, the current study validated the HSBS with one language group who were predominantly from two countries of origin (Syria and Iraq) which limits the generalizability of the findings especially as cultural factors are a large contributing factor to help-seeking beliefs. While a large proportion of the world’s refugees originate from Arabic-speaking countries, future studies should explore the psychometric properties of the HSBS across more diverse samples (UNHCR, 2023).

Third, while the high Omega and Alpha coefficients in this study suggest the scale is highly reliable, it may reflect the “reliability paradox” or the “attenuation paradox” whereby increasing reliability beyond an optimal point can paradoxically be associated with decreases in validity because highly related items represent only a narrow part of the construct (Loevinger, 1954). This can result in homogeneous items forming a separate factor; this is artefactual (Oltmanns & Widiger, 2018). Given our results support item convergence and divergence, this suggests items in the final scale are highly related but not necessarily redundant indicating that factors are genuine and not the artefactual result of homogeneous indicators binding tightly together. However, given the high degree of internal consistency, it is still important to consider that the HSBS may be a narrow representation of the entire construct of negative help-seeking beliefs. As such, future studies that aim to validate the HSBS in other samples should also comprehensively investigate item convergence and divergence to ensure results are replicated.

Finally, analyses exploring help-seeking intentions focused on PTSD symptoms given the prevalence of PTSD among refugees and to account for the role of trauma in endorsing help-seeking beliefs, which is one of the unique aspects of the refugee experience (Henkelmann et al., 2020). As other mental health disorders, including depression and anxiety, are highly prevalent in refugees compared with the general population, future research may wish to consider how symptom profiles may result in different HSBS subscales emerging as more prominent barriers in help-seeking (Blackmore et al., 2020). For example, people with anxiety disorders may be more likely to endorse greater fears of negative consequences due to future-oriented worry.

To our knowledge, this is the first study to develop and test a novel measure indexing negative help-seeking beliefs in refugees. This is important because while negative help-seeking beliefs are an established help-seeking barrier, unique refugee experiences (nature of trauma, displacement), and cultural factors may lead to specific beliefs that inhibit help-seeking among traumatized refugees. A scale that operationalizes and assesses for such beliefs is highly useful for a number of practical and theoretical reasons. First, the HSBS has the potential to be used among service providers to screen for potential negative perceptions of the help-seeking process. This allows clinicians to identify clients at risk of drop-out and to proactively resolve any misconceptions or fears a client may have. Given the observed relationship between self-stigma and help-seeking beliefs, using the scale to identify and target negative help-seeking beliefs may also have the added benefit of reducing self-stigma. A client’s responses on the scale also allows service providers to develop insight into their client’s fears and beliefs, which is important in building a culturally competent workforce and in turn increasing refugees’ positive experiences with mental health services (Xin, 2020). From a research perspective, the scale can be used as a foundation to measure the construct in other refugee populations by adapting and validating it for the specific cultural context. As discussed earlier, it also allows for investigation into longitudinal relationships between help-seeking beliefs and associated constructs, such as self-stigma, to further understand how help-seeking barriers interact over time. Understanding the nature of these beliefs represents a critical step to reducing the treatment gap and increasing uptake of mental health services among refugees with psychological disorders.
